# Antidiabetic potential of *Lavandula stoechas* aqueous extract: insights into pancreatic lipase inhibition, antioxidant activity, antiglycation at multiple stages and anti-inflammatory effects

**DOI:** 10.3389/fphar.2024.1443311

**Published:** 2024-10-30

**Authors:** Amal Elrherabi, Rhizlan Abdnim, El Hassania Loukili, Abdelouahid Laftouhi, Fatima Zahra Lafdil, Mohamed Bouhrim, Ramzi A. Mothana, Omar M. Noman, Bruno Eto, Abderrahim Ziyyat, Hassane Mekhfi, Abdelkhaleq Legssyer, Mohamed Bnouham

**Affiliations:** ^1^ Laboratory of Bioresources, Biotechnology, Ethnopharmacology, and Health, Faculty of Sciences Mohammed First University, Oujda, Morocco; ^2^ Euromed University of Fes, UEMF, Fes, Morocco; ^3^ Laboratory of Electrochemistry, Modeling and Environment Engineering (LIEME), Sidi Mohamed Ben Abdellah University, Faculty of Sciences Fes, Fes, Morocco; ^4^ Biological Engineering Laboratory, Faculty of Sciences and Techniques, Sultan Moulay Slimane University, Beni Mellal, Morocco; ^5^ Laboratories TBC, Laboratory of Pharmacology, Pharmacokinetics and Clinical Pharmacy, Faculty of Pharmacy, University of Lille, Lille, France; ^6^ Department of Pharmacognosy, College of Pharmacy, King Saud University, Riyadh, Saudi Arabia

**Keywords:** Lavandula stoechas, type 2 diabetes, antioxidant, pancreatic lipase, antiinflammatory, glycation

## Abstract

**Background:**

With the increasing global prevalence of type 2 diabetes (T2D) and obesity, there is a pressing need for novel therapeutic interventions. *Lavandula stoechas,* a medicinal plant traditionally used for various ailments, holds promise as a potential agent for T2D management, particularly in Morocco, where it is commonly used to treat diabetes. This study aims to evaluate the pharmacological potential of *L. stoechas* aqueous extract (AqLs) by assessing its lipase inhibition antioxidant and anti-inflammatory activities, identifying phenolic compounds, and examining its efficacy in reducing diabetic complications.

**Methods:**

The pharmacological potential of *L. stoechas* aqueous extract was investigated using *in vitro* assays. The inhibitory effect on pancreatic lipase, antioxidant power (FRAP), and anti-inflammatory activity (albumin denaturation method) was assessed. High-performance liquid chromatography (HPLC) analysis identified phenolic compounds. Additionally, albumin glycation was evaluated by estimating fructosamine, carbonyl groups, and amyloid β-structures to assess efficacy in mitigating diabetic complications.

**Results:**

The extract demonstrated concentration-dependent inhibition of pancreatic lipase (IC_50_ = 0.132 ± 0.006 mg/mL), potent antioxidant activity (IC_50_ = 604.99 ± 1.01 μg/mL), and dose-dependent anti-inflammatory effects (IC_50_ = 207.01 ± 34.94 mg/mL). HPLC analysis revealed phenolic compounds: naringin (38.28%), syringic acid (25.72%), and cinnamic acid (15.88%) were the most abundant, with 4-hydroxybenzoic acid, hydrated catechin, and catechin ranging from 9.60% to 5.24%, and p-coumaric acid (1.73%). Furthermore, the extract inhibited albumin glycation and fructosamine production, suggesting efficacy in mitigating diabetic complications.

**Conclusion:**

These findings highlight the multifaceted pharmacological potential of *L. stoechas* aqueous extract in T2D management, suggesting that this plant can be highly beneficial for diabetic individuals.

## 1 Introduction

With the rising prevalence of type 2 diabetes (T2D) and ongoing debates surrounding its multifaceted origins, emerging evidence underscores the pivotal role of lifestyle factors as significant predisposing elements. Both type 2 diabetes (T2D) and obesity are marked by chronic inflammation and insulin resistance (IR), and persistently elevated oxidative stress (OS) (Vlassara and Uribarri, 2014). In diabetes, there is typically an upsurge in free radical production alongside compromised antioxidant defenses, leading to cellular damage through mechanisms like the formation of advanced glycation end products (AGEs) (Khalid et al., 2022). Protein glycation leads to a partial loss of protein functionality and is a significant consequence of prolonged elevated blood sugar levels (Yamagishi and Matsui, 2010). It begins with reducing sugars (such as glucose, fructose, and pentose) interacting with available amino groups on proteins, forming schiff bases, fructosamine, and Amadori products. In the middle stage, these products degrade into carbonyl compounds like deoxyglucosones, glyoxal, and methylglyoxal. The final stage generates irreversible AGEs. Research suggests that interactions between glycated proteins, AGEs, and their receptors produce reactive oxygen species (ROS), triggering oxidative stress and inflammation in blood vessels. These processes contribute significantly to diabetes-related complications (Goh and Cooper, 2008). Individuals with diabetes exhibit considerably higher levels of oxidative stress compared to healthy individuals. This increased oxidative stress is often associated with lower serum concentrations of vitamin C and antioxidant enzymes such as glutathione peroxidase, superoxide dismutase, and catalase.

Additionally, there is an elevation in AGEs, protein carbonyls, conjugated dienes, and malondialdehyde levels (Martín-Gallán et al., 2003). Inflammation is a protective response initiated by the body in reaction to external triggers, characterized by localized redness, increased warmth, swelling, and discomfort. This process involves increased blood circulation and enhanced blood vessel permeability, accumulating fluids and inflammatory agents (such as ROS, eicosanoids, and cytokines) in the affected tissue. To counteract and suppress inflammation, the body releases cytokines that inhibit pro-inflammatory signaling pathways through feedback mechanisms, aiming to maintain homeostasis and ensure tissue wellbeing (Deretic and Klionsky, 2018).

One of the significant problems of type 2 diabetes is hyperlipidemia. Excess weight and obesity significantly elevate the risk for various chronic ailments, notably diabetes mellitus (Trigueros et al., 2013). Human pancreatic lipase (HPL), is the main enzyme that breaks down dietary fats. It primarily breaks down triglycerides into glycerol and free fatty acids. However, inhibiting these enzymes can slow digestion, decrease absorption, and lower postprandial blood sugar levels. Thus, inhibiting pancreatic lipase (PL) and reducing lipid absorption are promising approaches for treating obesity (Slanc et al., 2009). Despite advances in anti-hyperglycemic and antidiabetic medications, diabetes remains a global problem due to the high number of deaths it causes. While novel therapeutic compounds like insulin and oral hypoglycemic agents are available, their routine use is linked with various adverse effects (Bailey, 2008). Medicinal plants play a significant role in managing diverse illnesses, including diabetes. In Morocco, the use of over 100 medicinal plants for diabetes treatment has been documented. These plants' antidiabetic and antihyperglycemic activity is attributed to active compounds like polyphenols and terpenoids (Bnouham et al., 2002). *Lavandula stoechas* (Lamiaceae) is traditionally used in Morocco for diabetes treatment (Bousta and Farah, 2020). Although widely cultivated for various purposes (Sierra et al., 2009). It is particularly valued for its medicinal properties in Morocco, especially in the oriental region. At the same time, the essential oils of *Lavandula stoechas* have been extensively studied for their pharmacological properties (Bouyahya et al., 2017; Messaoud et al., 2012; Benabdelkader et al., 2011). In Morocco, *L. stoechas* is traditionally used to treat rheumatoid arthritis, nephrotic syndrome, and as an antispasmodic, as well as to alleviate pain and inflammation. Additionally, it is employed for its antidiabetic effects in the form of a decoction or infusion (Yassine et al., 2016). This genus is rich in phenolic compounds, including protocatechuic, ferulic, caffeic, rosmarinic, and chlorogenic acids, as well as pinocembrin, pinobanksin, quercetin, and luteolin (Lee et al., 2011).

Although studies have confirmed the antidiabetic properties of *L. stoechas*, primarily attributed to its essential oil, we attempted to demonstrate in our laboratory the antidiabetic effect of the most commonly used traditional extract, which is the aqueous extract of *L. stoechas*, using multiple methods (Elrherabi et al., 2023). To strengthen these findings, this study aimed to explore the potential of the aqueous extract of *L. stoechas* (AqLs) in preventing diabetic complications, particularly by examining its anti-glycation properties. The research evaluated various glycation markers at different stages: fructosamines for the initial stage, protein carbonyls for the intermediate stage, and AGEs for the final stage. Additionally, the study assessed albumin aggregation using amyloid-specific dyes like Congo red. It also aimed to identify the chemical profile, antioxidant activity (FRAP), antidenaturation effects on albumin, and the antihyperglycemic effect by inhibiting pancreatic lipase. We chose to work on this plant because it is widely used in oriental Morocco to treat diabetes as a decoction or infusion. However, most studies have focused on the plant’s essential oil. Therefore, we aimed to confirm the traditional use of its aqueous extract scientifically.

## 2 Materials and methods

### 2.1 Reagents and chemicals

P-nitrophenyl palmitate (pNPP), porcine pancreatic lipase, chicken egg albumin, aminoguanidine (AG), orlistat, trichloroacetic acid, diclofenac sodium, and Congo red were sourced from sigma-aldrich, Germany. Thiobarbituric acid (TBA) was acquired from Biosystems, Spain. PBS (phosphate-buffered saline), acetonitrile, potassium ferrocyanide (K_3_Fe (CN)_6_), and BSA (bovine serum albumin) were procured from sigma-aldrich, France.

### 2.2 Plant extraction

*Lavandula stoechas* specimens were initially sourced from Tafoughalt (Oriental Morocco) at coordinates 34.805497, −2.406913 during November 2023. The specimen was deposited in the herbarium of the faculty of sciences at Mohamed First University, Oujda, Morocco, and verified by a botanist under reference number HUMPOM77. To prepare the *L. stoechas* powder, the plant’s aerial parts were meticulously cleansed with distilled water to remove any impurities, followed by thorough drying at 40°C in an oven. Subsequently, the cleaned plant material was finely chopped. For extraction, 80 g of the prepared plant material was boiled in 800 mL of distilled water for 20 min using the decoction method. The resulting aqueous extract was filtered and then subjected to dehydration at 40°C to obtain the desired powdered Form of the plant. The extraction process yielded approximately 100 mg/g dry weight of the plant material. This rigorous process ensured the purity and integrity of the *L. stoechas* extract for subsequent analyses and experiments.

### 2.3 High-performance liquid chromatography (HPLC) analysis

The analysis of phenolic compounds within the aqueous extract of *L. stoechas* was determined by HPLC using an Agilent 1200 (Agilent Technologies, Palo Alto, CA, United States) coupled to a diode array UV detector (Bruker, Germany). Each extract (20 μL) was injected into a Zorbax XDB-C18 (5 μm porosity, 250 × 4.6 mm; Agilent Technologies Series 1100 system (Palo Alto, CA, United States)) column equipped with a 4 × 3 mm C18 cartridge precolumn (Agilent Technologies) with the following elution gradient: 0–25 min, 20% B 25–30 min, 100% B, and 30–35 min, 20% B. The mobile phases used for sample elution were A (water/0.5% phosphoric acid) and phosphoric acid) and B (methanol) with a 1 mL/min flow rate. The separation was performed at a constant temperature of 40°C. Spectrophotometric detection was performed at 254 nm. Compounds were identified by their retention times and UV spectra with those of authentic with those of authentic standards. Data analysis followed a methodology distinct from that of Loukili et al. (Haddou et al., 2023). The chemicals were identified by comparing their retention periods and UV spectra to those of legitimate standards. The quantification (Table 1) was carried out using calibration curves with different concentrations of standards in ethanol (0.2, 0, 4, 0, 6, 0, 8, and 1 mg/mL). For each molecule, the detection limit (LOD) and quantification limit (LOQ) were established using the following formulas:

LOD = 3.3∗ (standard deviation of signal intensity from low − concentration sample/slope of the calibration curve),
LOQ = 10∗ standard deviation of signal intensity from low − concentration samples/ slope of the calibration curve).

**TABLE 1:** Limit of detection (LOD), the limit of quanti6cation (LOQ) and correlation coefficients obtained in samples by the HPLC-DAD method.

N°	Compounds	Concentration range (mg/mL)	Linear regression	r^a^	LOD^b^ (mg/mL)	LOQ^c^ (mg/ml)
1	Ferulic acid	0.2–1	Y = 278.43x+13.33	0.98	0.05	0.15
2	Catechin	0.2–1	Y = 340.67x+23.00	0.98	0.10	0.31
3	Catechine hydrate	0.2–1	Y = 378.37x+20.59	0.98	0.01	0.04
4	Syringic acid	0.2–1	Y = 545.33x+76.42	0.98	0.23	0.69
5	4-Hydroxybenzoic acid	0.2–1	Y = 904.22x+40.56	0.98	0.05	0.14
6	Naringin	0.2–1	Y = 798.96x+116.15	0.98	0.24	0.72
7	Cinnamic acid	0.2–1	Y = 498.96x+21.50	0.98	0.08	0.25
8	*p*-coumaric acid	0.2–1	Y = 148.15x+18.78	0.98	0.32	0.97

Values are expressed as mean standard deviation; (r2)^a^: correlation coefficient; LOD^b^: limit of detection; LOQ^c^: limit of quantification.

### 2.4 Ferric reducing/antioxidant power (FRAP)

This assay aims to evaluate the iron-reducing potential of different tested extracts. We gauged the ferric-reducing capability of the tested extract using the protocol delineated by Dehpour et al. (2009), This method allowed for the conversion of Fe^3+^ in the K_3_Fe (CN)_6_ complex to its reduced Form, Fe^2+^. We prepared various concentrations of the tested extract for the procedure (0.31; 0.65; 0.125; 0.25; 0.5; and 1 mg/mL). A total of 0.5 mL of the sample was combined with 1.25 mL of phosphate buffer (0.2 M, pH 6.6) and 1.25 mL of potassium ferrocyanide (K_3_Fe (CN)_6_) (1% w/v). The mixture underwent incubation at 50°C for 20 min. After cooling to room temperature, the reaction was halted by adding 1.25 mL of trichloroacetic acid (10% w/v). Subsequently, the mixture was centrifuged at 1200 g for 10 min (Hermle Z230H, Gosheim, Germany). A 1.25 mL portion of the resulting floating liquid was mixed with 1.25 mL of distilled water and 0.25 mL of ferric chloride (0.1%) solution. Absorbance was measured at 700 nm using a spectrophotometer (Hitachi U-2900/2910 UV-Vis spectrophotometer), with distilled water serving as a reference rather than the solution of the extract. Ascorbic acid served as the standard reference, and each measurement was repeated thrice. An elevation in absorbance signifies an augmentation in the reducing power of the extract under evaluation.

### 2.5 The *in vitro* inhibitory effect of pancreatic lipase

The assay for pancreatic lipase inhibition was adapted from the methodology outlined by Jaradat et al. (2019). With some modifications, The pancreatic lipase inhibition assay employed p-nitrophenyl palmitate (pNPP) as the substrate. The plant extract was dissolved in Tris HCl buffer (pH 8) to create a mother solution at a concentration of 1000 μg/mL, which was then serially diluted to concentrations of 0.125, 0.25, 0.5, and 1 mg/mL. Porcine pancreatic lipase solution was prepared by dissolving 10 mg of enzyme in 10 mL of Tris HCl buffer at pH 8. A mother solution of the p-nitrophenyl palmitate (pNPP) substrate was created by dissolving 0.19 g of pNPP in 10 mL of acetonitrile. A mixture was prepared to conduct the inhibition activity test by combining 0.1 mL of lipase at 1 mg/mL, 0.2 mL of the plant extract solution at different concentrations, and 0.7 mL of Tris HCl buffer at pH 8. The resulting mixture was incubated at 37°C for 15 min, followed by adding 0.1 mL of the substrate solution (pNPP) and further incubation for 30 min at 37°C. Absorbance was measured at a wavelength of 410 nm using a spectrophotometer. Orlistat served as a positive control, and the procedure was repeated for orlistat using the same concentrations. All experiments were conducted in triplicate, and the percentage inhibition was calculated using the provided equation.

$$\%\mathrm{of inhibition}=\left(\frac{A0-A}{A0}\right)\times 100$$

A_0_ denotes the enzyme activity (absorbance) of the reaction mixture without the inhibitor (control), while A represents the enzyme activity (absorbance) of the reaction mixture with the inhibitor. The IC_50_ value signifies the concentration of the plant extract needed to inhibit 50% of the enzyme activity. This determination involves plotting a graph of substrate concentration versus inhibition percentages. Regression analysis is then utilized to ascertain the concentration at which enzyme activity is reduced by 50% (Dehpour et al., 2009).

### 2.6 The glycation of albumin assay, *in vitro*

The glycation of albumin assay was conducted according to the methodology described by Bouchmaa et al. (2022), With minor adjustments, we prepared a solution of 100 mM potassium phosphate buffer (pH 7.4, containing 0.2% sodium azide), in which BSA (10 mg/ml) was dissolved. Then, 1 ml of the bovine serum albumin (BSA) solution was mixed with a 500 mM glucose solution. The aqueous extract of *L. stoechas* and aminoguanidine (AG) which served as a positive control, dissolved in PBS (phosphate-buffered saline), were then added to the mixtures, which were subsequently incubated at 37°C in the dark for 1 week. Using a spectrofluorometer (Horiba FluoroMax-4 fluorescence spectrophotometer), we measured the fluorescence of advanced glycation end products (AGEs), with excitation and emission wavelengths set at 355 nm and 460 nm, respectively. AG was employed as the positive control. The percentage of inhibition was computed using the following formula and expressed accordingly for each test.

#### 2.6.1 Estimation of fructosamine

To assess fructosamine production, we employed the methodology outlined by Ravan et al. (2016), for the thiobarbituric acid (TBA) assay. This approach incubated glycated BSA with TBA (0.05 M). Following a 20-min incubation in a hot water bath, the mixtures were cooled to room temperature, and the absorbance at 443 nm was measured. Fructosamine inhibition percentage was then calculated using the following equation:

$$\%\mathrm{Of inhibition}=\left(\frac{A0-A1}{A0}\right)\times 100$$

A_0_ denotes the absorbance measurement from the positive control group, while A_1_ represents the absorbance measured upon adding the extracted sample.

#### 2.6.2 Estimation of carbonyl groups

To quantify albumin carbonyl compounds, we utilized the method delineated by Safari et al., 2018 (Safari et al., 2018), with minor adjustments, glass tubes were filled with an equal volume (0.5 ml) of glycated samples and 2,4-dinitrophenylhydrazine (DNPH) (10 mM in HCl 2.5 M), then left to incubate at room temperature for 1 hour. UV absorption was measured at 365 nm, and the molar extinction coefficient of DNPH (365 nm = 21 mM per cm) was applied to calculate the carbonyl content. The calculation for the percentage of inhibition was carried out as follows:

$$\%\mathrm{of inhibition}=1-\left(\frac{A0-A1}{A0}\right)\times 100$$

A_0_ represents the absorbance measurement from the positive control group, whereas A_1_ represents the absorbance measured upon adding the extracted sample.

#### 2.6.3 Estimation of amyloid β-structures

To quantify the Congo red binding to β-amyloid aggregation in the samples, we mixed 100 μl of Congo red (100 mM solution in phosphate-buffered saline, 10% ethanol) with 0.5 ml of glycated albumin. Following a 20-min incubation period at room temperature, the mixture underwent absorbance measurement at a wavelength of 530 nm (Tupe et al., 2015). The percentage of inhibition was determined using the following equation:

$$\%\mathrm{of inhibition}=1-\left(\frac{A0-A1}{A0}\right)\times 100$$

A_0_ represents the absorbance measurement from the positive control group, while A_1_ represents the absorbance measured upon adding the extracted sample.

### 2.7 The anti-inflammatory test, *in vitro*

*In vitro* anti-inflammatory activity of *L. stoechas* L. using albumin denaturation method was employed following the procedures outlined by Williams et al. (2008), with minor adjustments, we conducted the albumin denaturation method. Initially, 0.8 mL of fresh chicken egg albumin (12.5%) was mixed with phosphate-buffered saline (PBS) at a pH of 6.4. Subsequently, 0.6 mL of various concentrations of the AqLs were added to achieve final concentrations of 50, 100, 200, 400, 800, and 1000 mg/mL. These mixtures were then incubated at 37°C for 20 min, followed by heating at 70°C for 5 min. After cooling, absorbances were measured at 660 nm using a spectrophotometer. Diclofenac sodium, at final concentrations ranging from 50 to 1000 mg/mL, was employed as a reference drug and underwent similar treatment for absorbance determination.

The percentage inhibition was computed using the formula:

$$\%\mathrm{Of inhibition}=\left(\frac{\text{Asample}-\text{Acontrol}}{\text{Acontrol}}\right)\times 100$$

A dose-response curve was constructed to establish the concentration required for 50% inhibition (IC50).

### 2.8 Statistical analyses

The data analysis was performed using Graph Pad Prism 5, with results presented as the mean ± standard deviation of the mean (SD). Statistical analysis was conducted using one-way ANOVA to determine significant differences among groups, followed by Tukey’s Multiple Comparison Test for *post hoc* analysis to identify specific differences between groups. A significance level of *p* < 0.05 was considered statistically significant.

## 3 Results and discussion

### 3.1 High-performance liquid chromatography HPLC

**FIGURE 1:**
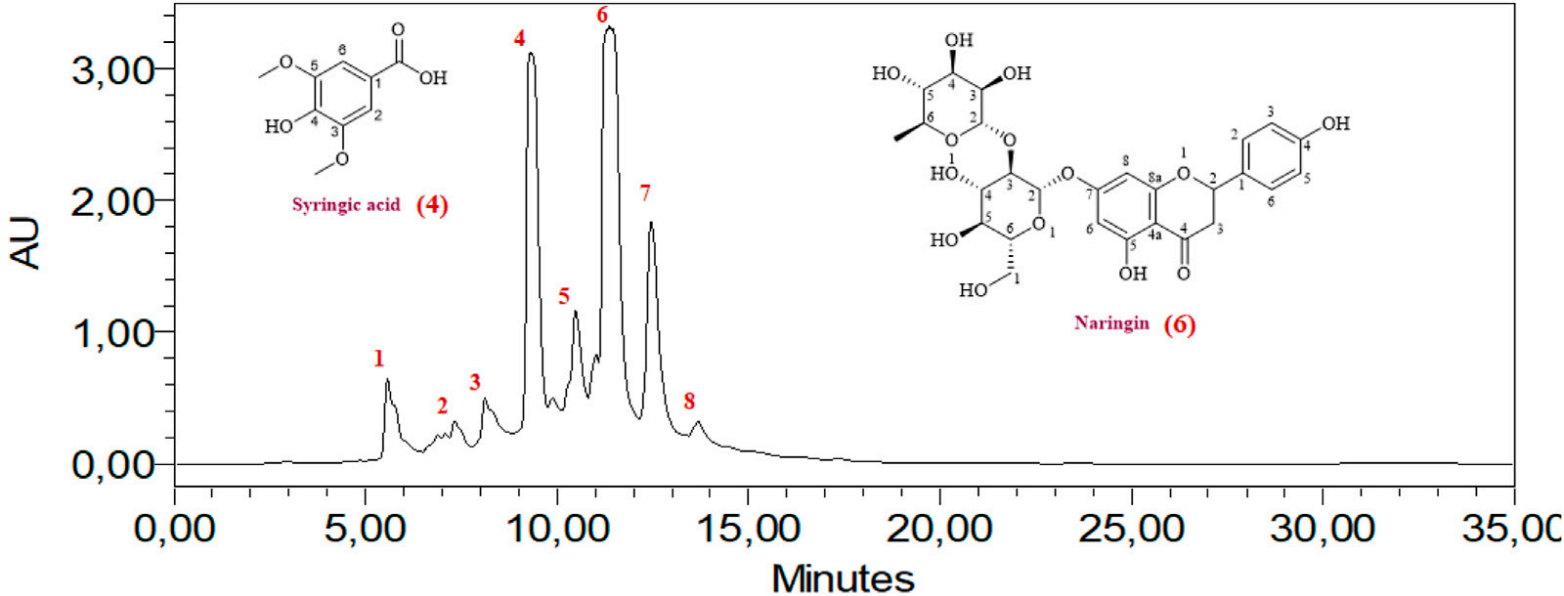
HPLC chromatogram of the aqueous extract of Lavandula stoechas L.

The HPLC analysis of phenolic compounds in Moroccan *L. stoechas* L. aqueous extract identified eight compounds (Table 2). The analysis of components found in aqueous extract was separated based on physical and chemical characteristics. This method can also detect the presence of certain compounds in the extract and quantify their amounts (Figure 1). Data analysis shows that naringin is the predominant phenolic compound, with 1290.32 ± 0.031 mg/100 g of the total area, thus indicating its abundance in the extract. Significant quantities of syringic and cinnamic acid are also observed, with area values of 1275.88 ± 0.455 and 864.41 ± 0.021 mg/100g, respectively. Additionally, 4-hydroxybenzoic acid, hydrated catechin, and catechin are present at moderate levels, ranging from 288.70 ± 0.636 to 563.21 ± 0.071 mg/100 g. P-coumaric acid, although present, appears in relatively lower amounts, with an area value of 310.34 ± 0.391 mg/100 g. Previous studies have established the plant’s chemical composition and shown that the biomolecules were extracted with various solvents and evaluated using spectroscopy and LC-MS. Flower extraction using aqueous acetone or methanol yielded the highest phenolic and flavonoid content. The examination and quantification of phenolic components of *L. stoechas* L leaves and stems in methanolic extracts revealed 21 elements, primarily Rutin (2133.00 μg/g of dry weight), Apigenin 7-glucoside (837.40 μg/g of dw), and Luteolin 7-glucoside (823.30 40 μg/g of dw) (Maouni et al., 2025). Another study by Ndhlala et al. found that LC-MS/MS analysis revealed that vanillic acid (125,596.66 μg/L) was the most abundant phytochemical in *L. stoechas* (Ndhlala et al., 2024). For Benyammi et al., the data analysis shows that naringénine is the predominant phenolic compound, with 16.85% of the total surface (Benyammi et al., 2023). Another study found flavones were a prominent class, constituting 52.35% of phenolic content. Key compounds identified included luteolin 7-O-glucuronide, rosmarinic acid, and apigenin 7-O-glucoside and 7-O-glucuronide. Further analysis via HPLC highlighted that phenolic acids, such as rosmarinic acid, caffeic acid, and quercetin, were more prevalent than flavonoids. Precisely, 8.347 mg/g of rosmarinic acid, 0.875 mg/g of caffeic acid, 1.019 mg/g of quercetin, and 0.472 mg/g of rutin were measured. These results indicate that phenolic acids are predominant over flavonoids in the extract. The study suggests that compounds like rutin, caffeic acid, and rosmarinic acid may significantly contribute to the antioxidant activity and therapeutic efficacy of *L. stoechas* (Ceylan et al., 2015; Contreras et al., 2018). High-Performance Liquid Chromatography/Time of Flight/Mass Spectrometry (HPLC-TOF/MS) was also used to analyze the phytochemical composition of the methanol extract. The main compound identified was rosmarinic acid, constituting 80.9% of the extract. The analysis also included quantifying phenolic compounds, organic acids, and flavonoids. The essential oil, obtained through steam distillation and analyzed by GC-MS, primarily contained camphor (48.1%) and fenchone (30.5%) (Karabagias et al., 2019).

**TABLE 2:** Quantitative phytochemical analysis of aqueous extract of *Lavandula stoechas*.

N°	Compounds	Retention time	Ls (mg/100 g) ±SD
1	Ferulic acid	5.58	512.76 ± 0.684
2	Catechin	7.33	247.24 ± 0.048
3	Catechine hydrate	8.12	563.21 ± 0.071
4	Syringic acid	9.13	1275.88 ± 0.455
5	4-Hydroxybenzoic acid	10.46	288.70 ± 0.636
6	Naringin	11.33	1290.32 ± 0.031
7	Cinnamic acid	12.46	864.41 ± 0.021
8	*p*-coumaric acid	13.69	310.34 ± 0.391

These results underscore the compositional profile of phenolic compounds in the AqLs extract, with naringin being the most abundant, followed by syringic acid and cinnamic acid. Previous studies have demonstrated the bioactive properties of these phenolic compounds. For instance, oral administration of naringin significantly reduced fasting blood glucose levels and HbA1c, suggesting its beneficial effects in preventing diabetic complications associated with impaired glucose metabolism (Ahmed et al., 2012). Syringic acid has been shown to stimulate pancreatic beta-cells, which is essential for insulin production and secretion. In alloxan-treated diabetic rats, syringic acid reversed abnormalities in glycoprotein component levels, increased plasma insulin levels, and restored the normal function of carbohydrate metabolism enzymes (Srinivasan et al., 2014). Cinnamic acid also demonstrates antidiabetic properties through various mechanisms, such as stimulating insulin secretion, enhancing the function of pancreatic β-cells, inhibiting gluconeogenesis, increasing glucose uptake, and improving glucose tolerance. Additionally, cinnamic acid possesses antioxidant, hepatoprotective, and anti-inflammatory effects (Savych et al., 2021).

In summary, our HPLC analysis confirms the presence of bioactive phenolic compounds in *L. stoechas* aqueous extract, aligning with previous research findings and highlighting their potential health benefits.

### 3.2 Effect on the reducing power of iron

Oxidative stress occurs due to an imbalance between the generation of oxygen-derived radicals and the antioxidant defense mechanisms within the body (Abdollahi et al., 2004). Several investigations have revealed a correlation between diabetes mellitus, heightened free radical formation, and diminished antioxidant capacity. This imbalance disrupts the equilibrium typically maintained within cells, leading to oxidative damage to proteins, lipids, and nucleic acids. Elevated oxidative stress is evident in both type 1 (insulin-dependent) and type 2 (non-insulin-dependent) diabetes (Naziroğlu and Butterworth, 2005). Diabetes exacerbates oxidative stress through various mechanisms. Glucose autoxidation is a key contributor to generating free radicals. Imbalances in cellular redox processes and decreased antioxidant defenses also play significant roles, characterized by lower cellular antioxidant levels and reduced activity of antioxidant enzymes. Elevated levels of pro-oxidants like ferritin and homocysteine are expected in diabetes.

**FIGURE 2:**
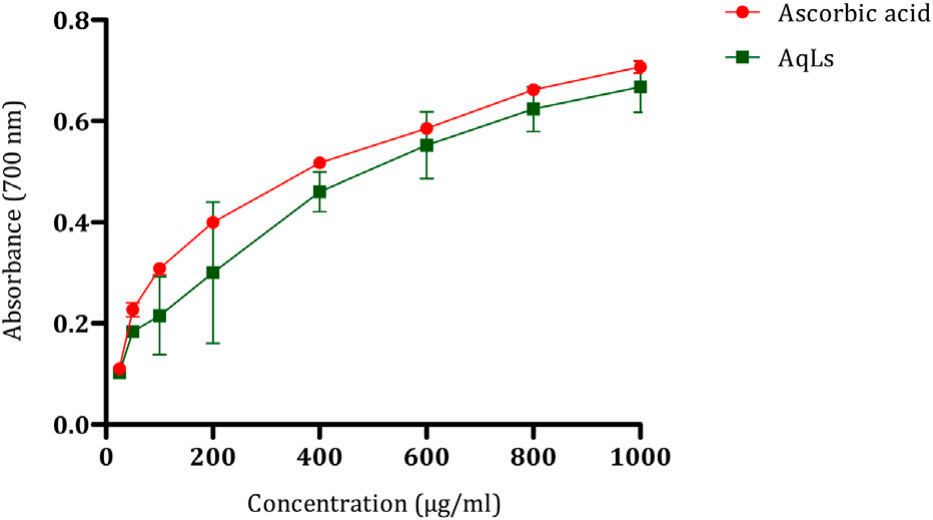
Antioxidant effect of AqLs on iron reduction (FRAP). The values are presented as means ± standard error of the mean (SEM) (n = 3). AqLs: Aqueous extract of L. stoechas, FRAP: Ferric Reducing Antioxidant Power.

Additionally, the interaction between AGEs and their receptors (RAGE) is crucial. AGE accumulation, initiated by glucose interaction with specific amino acids on proteins, leads to subsequent reactions. Protein glycation alters protein and cellular function, while AGE-RAGE binding modifies cell signaling, further enhancing free radical production (Penckofer et al., 2002). Oxidative stress is proposed as a standard pathway linking various mechanisms in the pathogenesis of diabetes complications (Shih et al., 2002). After demonstrating the antioxidant effect of the AqLs through multiple methods, such as beta-carotene bleaching assay and DPPH, in our previous study (Elrherabi et al., 2023), we also explored the antioxidant activity of *L. stoechas* using the iron reduction method, which involves the conversion of Fe^3+^ to Fe^2+^ in the presence of antioxidant components in the tested extract (Benzie and Devaki, 2018). The results, as illustrated in Figure 2, reveal that the antioxidant potential of the aqueous extract of *L. stoechas* is proportional to the concentrations, similar to that of ascorbic acid. An increase in optical density indicates a strengthening of the reducing power of the tested extract.

Comparing the effects of the plant and ascorbic acid, we observe that ascorbic acid has a median inhibitory concentration (IC_50_) of 552.1 ± 0.75 μg/ml, while the AqLs displays an IC_50_ of 604.99 ± 1.01 μg/ml. Although the reducing effect of the aqueous extract of *L. stoechas* is notable, ascorbic acid appears to demonstrate slightly higher efficacy in this specific antioxidant evaluation method. These results suggest that the plant has significant antioxidant potential, although ascorbic acid remains a notable reference in terms of antioxidant activity. The decrease in antioxidant activity of the plant extract towards ferric ions is linked to its phytoconstituents, including phenolic acids and flavonoids. These compounds act by disrupting the free radical chain, thereby influencing the antioxidant capacity.

Consequently, the redox potential of phenolic compounds is crucial in assessing antioxidant capability (Croft, 2016). The significant total phenolic content observed in the aqueous extract of *L. stoechas*, as demonstrated in a previous study, suggests the antioxidant potential of the extract (Elrherabi et al., 2023). The FRAP assay of the AqLs revealed a lower antioxidant potential than the standards BHT and BHA. In this assay, higher absorbance indicates greater ferric reducing antioxidant power. The antioxidant capacity of the plant extract is attributed to its phytoconstituents, such as phenolic acids and flavonoids, which can break the free radical chain. Therefore, the redox potential of these phenolic compounds plays a crucial role in determining the antioxidant capacity (Ceylan et al., 2015).

### 3.3 *In vitro* inhibitory effect on pancreatic lipase

**FIGURE 3:**
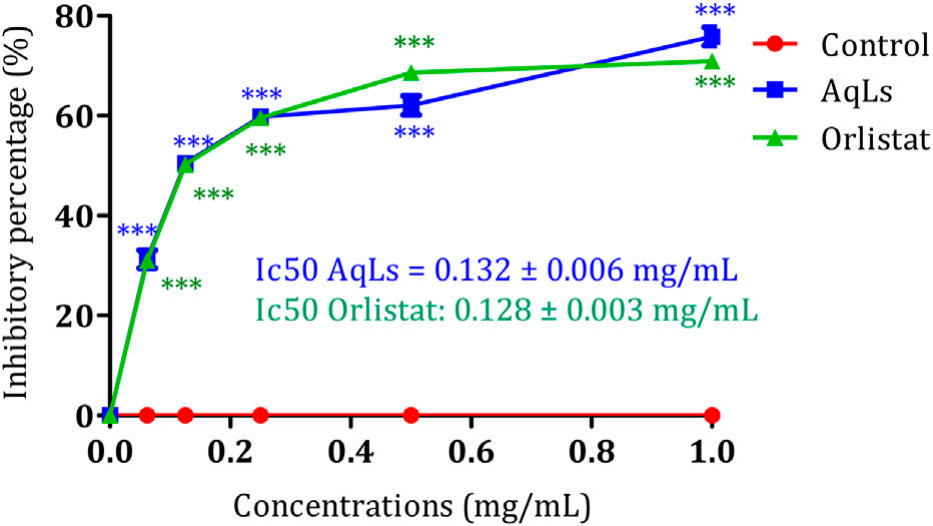
Inhibition activity on porcine pancreatic lipase by AqLs and Orlistat. The data is presented as mean ± standard deviation (n = 3). AqLs: Aqueous extract of L. stoechas, Ic50: half maximal inhibitory concentration.

Human pancreatic lipase (HPL), also known as pancreatic triacylglycerol lipase (EC: 3.1.1.3), is crucial for the digestion of dietary fats in the human digestive system (Kim et al., 2021). Studies have shown that HPL can hydrolyze 40%–70% of ingested triacylglycerides (TAGs) (Birari and Bhutani, 2007). Given its significance in lipid metabolism, inhibiting pancreatic lipase (PL) presents a promising avenue for developing treatments for obesity (Slanc et al., 2009). By reducing lipid absorption, inhibitors of PL offer potential therapeutic benefits in combating obesity. Additionally, targeting essential enzymes involved in carbohydrate digestion, such as α-amylase (1,4-alpha-D-glucan glucanohydrolase, EC: 3.2.1.1) and α-glucosidase (maltase-glucoamylase, EC: 3.2.1.20; EC: 3.2.1.3), offers potential benefits for managing diabetes and obesity by regulating postprandial glucose levels (Buchholz and Melzig, 2016). Figure 3 depicts the inhibitory impact of AqLs on pancreatic lipase activity using the p-nitrophenyl palmitate-based assay. The extract effectively suppressed porcine pancreatic lipase activity, showing a 75% inhibition rate at 1 mg/mL concentration. To gauge the potency of the inhibition, the IC_50_ value was computed, representing the concentration required to inhibit 50% of porcine pancreatic lipase activity. AqLs demonstrated an IC_50_ value of 0.132 ± 0.006 mg/mL. For comparison purposes, the IC_50_ value of the reference compound orlistat was measured at 0.128 ± 0.003 mg/mL. This establishes a benchmark for evaluating the inhibitory activity of the extract on pancreatic lipase. Breaking down dietary triglycerides is essential for their absorption, as intestinal enterocytes cannot directly absorb triglycerides. Instead, dietary triglycerides must be hydrolyzed into free fatty acids and monoacylglycerols before absorption (Mu and Høy, 2004). The initial digestion of triglycerides starts in the stomach, where pancreatic lipase releases approximately 15% of the fatty acids from triglycerides (Zhu et al., 2013). Diabetes often leads to dyslipidemia, a condition characterized by abnormal lipid and lipoprotein levels. This includes elevated triglycerides, reduced high-density lipoprotein (HDL) cholesterol, and a shift towards small, dense, low-density lipoprotein (LDL) particles, commonly referred to as diabetic dyslipidemia (Arca et al., 2012). Insulin resistance is a key factor contributing to this condition (Adiels et al., 2008). Elevated serum lipid levels in diabetes increase the risk of coronary heart disease (CHD) (Murase et al., 2008). The heightened serum lipid levels in diabetic individuals predominantly stem from the increased release of free fatty acids from peripheral fat stores. This occurrence is due to the inhibition of hormone-sensitive lipase by insulin. However, hormones like glucagon and catecholamines promote lipolysis, leading to pronounced hyperlipidemia characteristic of diabetes (Cryer, 2012). In alloxan-induced diabetes mellitus, increased blood glucose levels are frequently accompanied by increased serum cholesterol and triglycerides (Pari and Saravanan, 2002). This study demonstrated the inhibitory activity of the AqLs on pancreatic lipase for the first time, suggesting its potential as a therapeutic agent in managing obesity and dyslipidemia.

### 3.4 Antiglycation of albumin effect

In living organisms, sugars such as fructose and glucose can undergo non-enzymatic binding with proteins, resulting in structural and functional alterations. This process, termed glycation, contributes to the generation and buildup of advanced glycation end products (AGEs). Subsequently, the attachment of AGEs to receptors like RAGE leads to the breakdown of functional proteins and tissue damage. The buildup of AGEs further exacerbates the development and advancement of diabetic complications. Glycation is also associated with accelerated aging, a condition called glycation stress, which has recently gained attention (Ishioka et al., 2015). The combination of oxidative stress and free radical production exacerbates the situation, highlighting the urgency to identify novel compounds with anti-glycation and anti-oxidation properties (Ramdan et al., 2017). This study represents the first instance of demonstrating the anti-glycation effect of the AqLs across all three stages of the glycation process.

#### 3.4.1 Impact of AqLs on various phases of albumin glycation

The results presented in Table 3 demonstrate the significant inhibitory effect of AqLs on albumin glycation across various stages of glycation progression. In the early stage, represented by fructosamine (TBA assay), exhibited inhibition by AqLs with IC_50_ values of 0.41 ± 0.004 mg/mL, compared to 0.47 ± 0.008 mg/mL for AG (standard control). In the intermediate stage, markers such as protein carbonyl compounds (DNPH assay) were also inhibited, with IC_50_ values of 0.51 ± 0.005 mg/mL for AqLs and 0.46 ± 0.003 mg/mL for AG. Moreover, fluorescence analysis of advanced glycation end-products (AGEs) in later stages indicated the inhibitory properties of AqLs, with IC_50_ values of 0.48 ± 0.004 mg/mL for AqLs and 0.44 ± 0.004 mg/mL for AG, respectively. The inhibitory effect was dose-dependent, with the most effective inhibition observed at 1 mg/mL.

**TABLE 3:** The IC_50_ values for the inhibition of albumin glycation, determined through assessments involving TBA, DNPH, and Congo red tests, as well as the formation of AGEs, and antioxidant effect (FRAP), are presented in milligrams per milliliter (mg/mL).

	IC_50_				
	Antioxidant activity	Inhibitory activity of albumin glycation			
	FRAP (µg/mL)	TBA (mg/mL)	DNPH (mg/mL)	Congo red (mg/mL)	AGEs (mg/mL)
AqLs	604.99 ± 1.01 (a)	0.41 ± 0.004 (a)	0.51 ± 0.005 (a)	0.54 ± 0.004 (a)	0.48 ± 0.004 (a)
AG	—	0.47 ± 0.008 (a)	0.46 ± 0.003 (a)	0.46 ± 0.004 (a)	0.44 ± 0.004 (a)
AA	552.1 ± 0.75 (a)	—	—	—	—

AqLs: Aqueous extract of *Lavandula stoechas,* AG: aminoguanidine, AA: ascorbic acid, FRAP: ferric reducing antioxidant power, TBA: thiobarbituric acid, DNPH: 2,4-dinitrophenylhydrazine, AGEs: advanced glycation end products. Statistical designations in the table were determined using Tukey’s Multiple Comparison Test. Statistical significance was defined at *p* < 0.05. (a) indicates no significant difference between AqLs and AG, or AA.

#### 3.4.2 The impact of AqLs on β-aggregation during glycation

Glycation is a pivotal mechanism that alters protein conformation by increasing amyloid cross β-structures, which is essential in protein aggregation (Taghavi et al., 2017). This aggregation is associated with amyloidosis, a process linked to several chronic diseases, including diabetes (Soros et al., 2010). Our investigation aimed to evaluate how AqLs can impede the aggregation of glycated albumin using Congo red amyloid markers.

**FIGURE 4:**
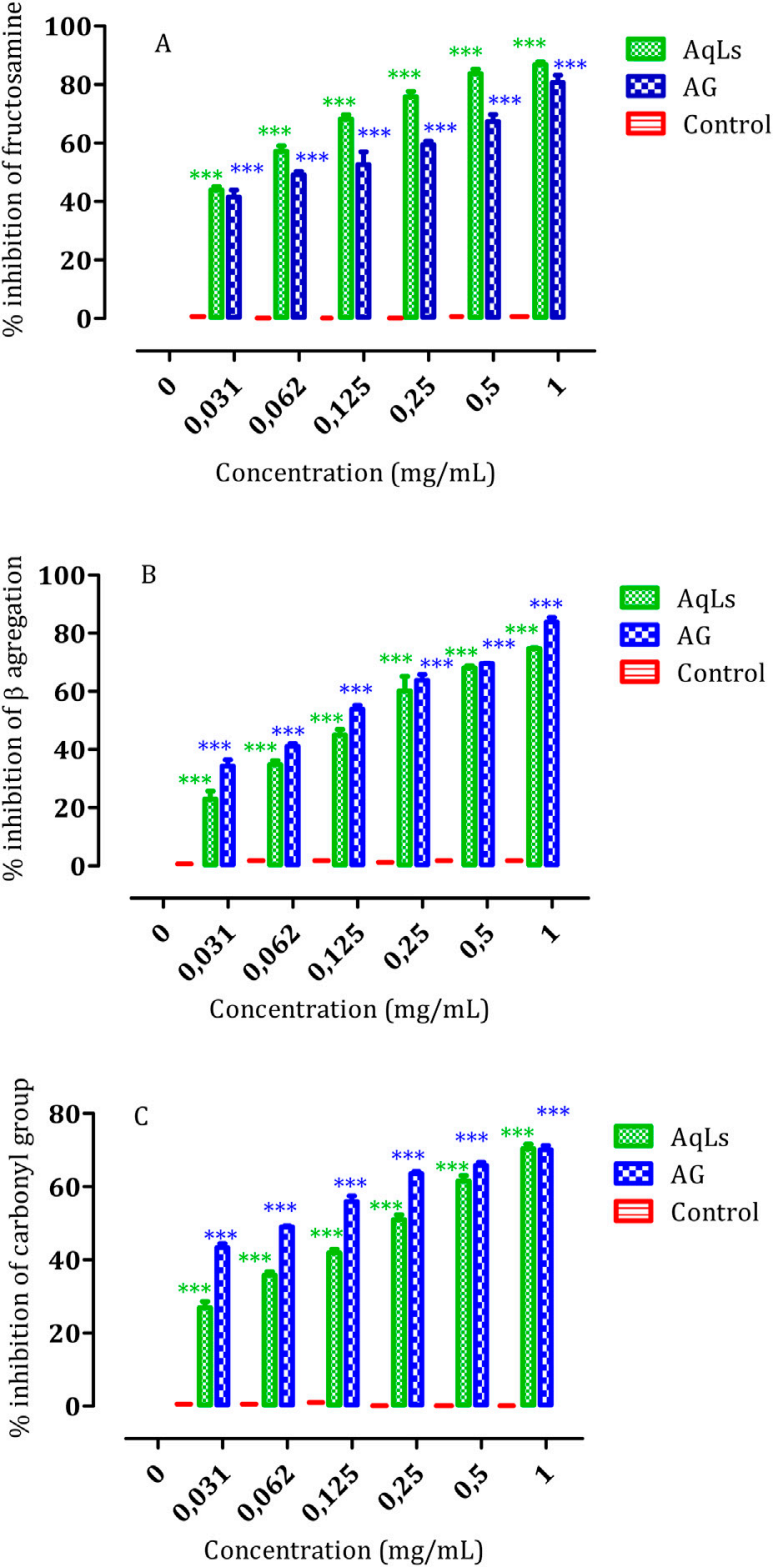
The influence of AqLs and AG (1 mg/ml) on modifications in albumin glycation, including **(A)** Fructosamine inhibition, **(B)** β-aggregation inhibition, and **(C)** Protein carbonyls inhibition, was investigated. The results, presented as means (n = 3), indicate the percentage of inhibition relative to the positive control. Significant differences are denoted as *** (*p* < 0.001). AqLs: Aqueous extract of L. stoechas, AG: Aminoguanidine.

Figure 4 illustrates the robust inhibitory effect of AqLs on amyloid formation. The IC_50_ values presented in Table 2 highlight the potent inhibitory activity of AqLs on amyloid cross β-structures, with an IC_50_ value of 0.54 ± 0.004 mg/mL compared to 0.46 ± 0.004 mg/mL for the standard control, aminoguanidine (AG). These findings align with previous studies that demonstrated the effectiveness of lavender extracts in inhibiting protein glycation and aggregation (Ho et al., 2014).

The study results show significant inhibition of albumin glycation across all three stages of glycation progression by AqLs. The suppression of fructosamine formation (early glycation products), protein carbonyl compounds (intermediate stage markers), and advanced glycation end-products (AGEs) (later stages) was notable. The inhibitory activity of AqLs was comparable to AG, indicating its potential as an effective anti-glycation agent. The inhibition mode displayed a dose-dependent relationship, with the optimal dosage observed at 1 mg/mL for each extract.

Furthermore, the study assessed the effect of AqLs on β-aggregation during glycation. The results revealed that AqLs effectively inhibited the aggregation of glycated albumin, as shown by the significant inhibition observed alongside the amyloid marker. The IC_50_ values further corroborated the inhibitory activity of AqLs on amyloid cross β-structures, emphasizing its potential as a therapeutic agent for preventing protein aggregation-related disorders.

Overall, these findings suggest that AqLs possess promising anti-glycation properties, which could have implications for preventing and managing conditions associated with abnormal protein glycation and aggregation, such as diabetes. These results are consistent with other research highlighting the therapeutic potential of natural compounds in mitigating the adverse effects of glycation and oxidative stress.

### 3.5 The anti-inflammatory activity

Protein denaturation is extensively documented and often occurs due to inflammatory processes, particularly in conditions like arthritis. Consequently, using agents capable of preventing protein denaturation holds promise for developing anti-inflammatory drugs. Various anti-inflammatory drugs have demonstrated a dose-dependent ability to inhibit heat-induced protein denaturation (Shallangwa et al., 2013; Shallangwa et al., 2015). Protection or inhibition against protein denaturation, which constitutes the primary mechanism of action of NSAIDs (non-steroidal anti-inflammatory drugs), significantly contributes to their anti-inflammatory activity (Shallangwa et al., 2013). Using *in vitro* methods for studying anti-inflammatory activities offers distinct advantages over animal experimentation in pharmacological research, addressing ethical concerns and providing alternative avenues where suitable methods are available (Shallangwa et al., 2015). The results concerning the anti-denaturation of proteins by the AqLs are outlined in Table 4. These results indicate that the ability to prevent protein (albumin) denaturation is correlated with the concentration of the aqueous extract of *L. stoechas*, which exhibits significant inhibition activity against albumin denaturation, as evidenced by an IC_50_ value of 207.01 ± 34.94 mg/mL. This inhibition is comparable to diclofenac (standard drug), which also shows an IC_50_ value of 207.966 ± 5.488 mg/mL. This study, for the first time, confirms the anti-inflammatory activity of the aqueous extract of *L. stoechas* through its inhibition of protein denaturation. Protein denaturation, a process where proteins lose their structure due to external stress or compounds, is a marker for inflammatory and arthritic diseases. The capacity of flavonoids to inhibit protein denaturation supports their anti-inflammatory properties. The flavonoid-rich extract effectively inhibited albumin denaturation in this study, demonstrating its potential as an anti-inflammatory agent (Enechi et al., 2020).

**TABLE 4:** IC_50_ values of the inhibition of denaturation by AqLs (mg/mL).

	IC_50_ (mg/mL)
AqLs	207.01 ± 34.94 (a)
Diclofenac	207.966 ± 5.488 (a)

(a) indicates no significant difference between AqLs and Diclofenac.

## 4 Conclusion

The present study highlights the potential therapeutic implications of *L. stoechas* aqueous extract (AqLs) in managing metabolic disorders such as obesity, diabetes, and associated complications. Through comprehensive *in vitro* investigations, we demonstrated the inhibitory effect of AqLs on pancreatic lipase activity, indicating its potential as a candidate for developing anti-obesity agents.

Furthermore, the antioxidant properties of AqLs were evidenced by their ability to reduce iron ions, suggesting their role in combating oxidative stress, a hallmark of various metabolic diseases, including diabetes. The phytochemical analysis revealed the presence of several bioactive compounds in AqLs, mainly phenolic compounds such as naringin, syringic acid, and cinnamic acid. These compounds are known for their antioxidant and anti-inflammatory properties, further supporting the observed pharmacological activities of the extract.

Moreover, our study elucidated the anti-glycation effects of AqLs across multiple stages of albumin glycation, highlighting its potential in mitigating the harmful effects of protein glycation associated with diabetes and aging. The inhibitory activity of AqLs on β-aggregation during glycation suggests its role in preventing protein conformational changes and related disorders such as amyloidosis.

Overall, the findings presented herein underscore the pharmacological potential of AqLs as a valuable natural resource for developing therapeutic agents targeting metabolic disorders. These results lay the groundwork for future studies to further explore the clinical applications of AqLs in preventing and managing metabolic diseases.

## Data Availability

The raw data supporting the conclusions of this article will be made available by the authors, without undue reservation.
